# Developing a complex intervention whilst considering implementation: the TANDEM (Tailored intervention for ANxiety and DEpression Management) intervention for patients with chronic obstructive pulmonary disease (COPD)

**DOI:** 10.1186/s13063-021-05203-x

**Published:** 2021-04-06

**Authors:** Liz Steed, Karen Heslop-Marshall, Ratna Sohanpal, Sarah Saqi-Waseem, Moira Kelly, Hilary Pinnock, Stephanie Taylor

**Affiliations:** 1 Institute for Population Health Sciences, Barts and the London School of Medicine and Dentistry, Queen Mary University of London, 58 Turner Street, London, E1 2AB UK; 2 Newcastle upon Tyne NHS Hospitals Foundation Trust, Chest Clinic, New Victoria Wing RVI Hospital, Queen Victoria Road, Newcastle upon Tyne, NE1 4LP UK; 3 University College London Hospitals NHS Foundation Trust, 250 Euston Road, NW1 2PG London, UK; 4 Allergy and Respiratory Research Group, Usher Institute of Population Health Sciences and Informatics, Doorway 3, Medical School, Teviot Place, Edinburgh, EH8 9AG UK

**Keywords:** Depression, Anxiety, Cognitive behavioural therapy (CBT), Self-management, Chronic obstructive pulmonary disease (COPD), Implementation, Intervention development

## Abstract

**Background:**

Guidelines now call for a thorough and comprehensive description of the development of healthcare interventions to aid evaluation and understanding of the processes of change. This was the primary aim of this study but we also recognised that effective interventions are commonly not implemented in clinical practice. It is suggested that insufficient attention is given to the implementation process at the development phase of interventions. This study outlines the 5 step iterative process we adopted for considering both implementation and effectiveness issues from the outset of intervention development. We use the development of a complex intervention Tailored intervention for ANxiety and DEpression Management (TANDEM) in patients with chronic obstructive pulmonary disease to illustrate this process.

**Methods:**

Intervention development built upon the Medical Research Council framework for developing complex interventions and the person-based approach for development of behavioural interventions. Building an expert team, specifying theory, qualitative data collection and pre-piloting were all critical steps in our intervention development and are described here.

**Results:**

Contact with experts in the field, and explicitly building on previous work, ensured efficiency of design. Qualitative work suggested guiding principles for the intervention such as introducing mood in relation to breathlessness, and providing flexible tailoring to patients’ needs, whilst implementation principles focused on training selected respiratory professionals and requiring supervision to ensure standards of care. Subsequent steps of intervention development, pre-piloting and intervention refinement led to an intervention that was deemed acceptable and if successful will be ready for implementation.

**Conclusions:**

The TANDEM study was developed efficiently by building on previous work and considering implementation issues from the outset, with the aim that if shown to be effective it will have more rapid translation in to the health care system with accelerated patient benefits.

**Trial registration:**

ISRCTN ISRCTN59537391. Registered on 20 March 2017. Protocol version 6.0, 22 April 2018.

**Supplementary Information:**

The online version contains supplementary material available at 10.1186/s13063-021-05203-x.

## Contributions to the literature

- We describe a five-step iterative process to developing the TANDEM intervention considering both effectiveness and implementation strategies throughout.
- We engaged an expert team and built upon previous work to ensure an efficient approach to intervention design.
- The approach builds on and integrates the Medical Research Council complex intervention framework and the person-based approach to intervention development and includes both health care professional and patient input from the outset.
- The study illustrates how testing of the intervention as a whole in the development phase can be used to promote effectiveness and develop implementation strategies prior to formal piloting of the trial processes.

## Background

In recent decades, the science of developing complex interventions has made significant strides forwards with publication of guidelines and frameworks [1–3]. Recognised methods for categorising and describing interventions have become available, e.g. the Template for Intervention Description and Replication checklist TIDieR [4], the Workgroup for Intervention Development Recommendations (WIDER) [5] and, most recently, for describing intervention development GUIdance for the reporting of Intervention Development GUIDED [6]. These methods are recommended with a view to enabling replication by healthcare services. Despite this, there is commonly a gap between an intervention being shown to be clinically and cost effective and its implementation into practice [7, 8]. Reasons for this will be multifactorial but include organisational factors not being considered sufficiently at the outset or a design which does not lend itself to delivery as intended (fidelity) in clinical practice. This is increasingly leading to multiple research phases where an effectiveness trial is followed by development and evaluation of implementation strategies [9, 10].

This trajectory of stepwise development is costly and inefficient and may delay attainment of beneficial health outcomes. Recent approaches that may improve the process of complex intervention development include building and improving upon existing interventions, using a collaborative approach to intervention development and considering implementation issues (including enhancing fidelity) at the *outset* [11].

The current article uses the development of a complex intervention, TANDEM, for patients with moderate to very severe chronic obstructive pulmonary disease (COPD) and mild to moderate anxiety and/or depression, to illustrate how we built on existing innovations, involved expert stakeholders in a collaborative team approach and considered implementation throughout the process. Our dual aims were to optimise effectiveness and also implementation of the intervention, if it is shown to be effective.

### Managing chronic obstructive pulmonary disease (COPD)

COPD has a global prevalence of 11.7% in adults aged over 30 years [12] and is associated with substantial morbidity and mortality [13] with indications that it will be among the top three causes of death by 2030 [14]. Progressive reduction in lung function and increased breathlessness affect the physical, social and emotional worlds of patients [15]. People with COPD typically have multi-morbidity, including psychological conditions [16–18], such as anxiety and depression, which have a major influence on quality of life [19, 20]. Anxiety is reported across all ranges of COPD severity, with cited prevalence ranging from 10 to 50% depending on definitions and populations studied [21, 22]. The prevalence of depression (on average around 30% of all COPD patients [21, 23]) increases with the severity of COPD [24]. Importantly, people with COPD and anxiety/depression experience more exacerbations, more frequent and longer hospital admissions and reduced survival [22, 25–28].

**Fig. 1:**
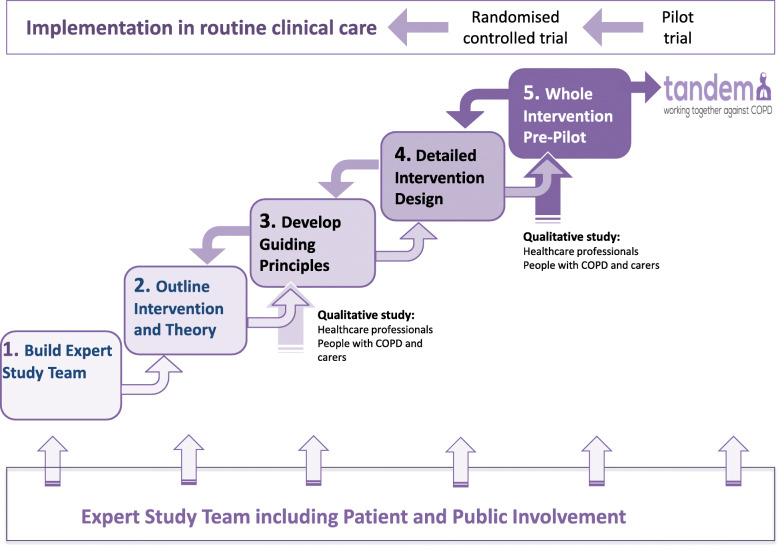
Schema for TANDEM Intervention Development Process

Pulmonary rehabilitation (PR) is an evidence-based, guideline-recommended intervention for the management of COPD [29], which improves functional capacity, psychological well-being and quality of life [29–31]. However, uptake and reach of PR is less than optimal [32] as practitioners under-refer, and patients decide not to, or are unable to, attend, or complete, their PR course [33] which may be in part due to co-existing anxiety and/or depression [34]. Psychological interventions using a cognitive behaviour therapy (CBT) approach either alone or as a component of PR have shown promise [30, 35, 36]. However, the limited evidence, to date, has not considered implementation issues such as reach or the practicalities of workforce delivery. TANDEM was developed to address this research gap and specifically to design an implementable, cognitive behavioural approach (CBA) intervention, delivered in tandem with PR, aiming to both improve symptoms of anxiety and depression and increase uptake and completion of PR, which itself further improves psychological well-being. The protocol for the TANDEM trial is published [37]. By CBA, we recognise that our intervention does not deliver full CBT but rather draws on the underlying theory to deliver an approach based on CBT. From the outset the process of developing the intervention was understood to be complex, and we kept an open mindset to different guidance and methodologies that we would need. This has subsequently been distilled into a five-step iterative process which is described below and illustrated in Fig. 1. Throughout these steps, we also worked closely with our PPI co-applicant, who had been instrumental in designing the original grant proposal and remained as an expert by experience throughout the development of the intervention.

## Methods

### Step one—Building an expert study team

Development of TANDEM arose out of a commissioned funding bid for a tailored psychological intervention ‘combined with physical retraining’ for individuals with anxiety and or depression and moderate to severe COPD. A review cited within the commissioning brief [36] suggested there was synergy between psychological approaches and physical activity in improving COPD. Proposals were requested to meet this need. In order to design and deliver such an intervention, the first step was building a multidisciplinary team with relevant expertise. The expertise that was deemed necessary included individuals with experience in respiratory medicine, pulmonary rehabilitation, cognitive behaviour therapy in COPD, self-management support in long term conditions, educationalists, health and clinical psychology, trials and research methodology, qualitative methodology and the lived experience of COPD (PPI). Individuals who the principal investigators (SJCT and HP) knew in each of these field were invited to participate in the expert team.

The expert team met at each stage of the intervention development process to suggest content, consider theory, review feedback and revise the intervention.

### Step two—Developing an outline of the intervention and consideration of theory

Based on the experience of the expert team, suggestions for an initial outline intervention (see supplementary file 1) were made. The theory underpinning these previous interventions was then identified.

Theory use was recommended at multiple levels with multiple objectives (e.g. to guide the intervention development process, to inform the patient facing element of the intervention, to inform training in delivery of the facilitator facing intervention). Selection of specific theory was guided by those with which the expert team had experience and which were supported by the literature. Implementation theories were not considered at this point as the plan was to initially understand implementation issues (in line with a hybrid type 1 approach [38] and then be directed to an appropriate theory as part of the process evaluation (Kelly M, SL, Sohanpal R, Pinnock H. Taylor S. The TANDEM trial: protocol for the process evaluation of a randomised trial of a complex intervention for anxiety or depression in people living with chronic obstructive pulmonary disease. Under review). 

Given the theoretical complexity of the intervention, we developed a programme theory *and* a logic model showing how we envisaged the different approaches would work together and complement each other in the overall TANDEM intervention.

### Step three—Qualitative research to understand participant and implementation needs and develop guiding principles

Building on the expert teams past experience, and materials and theory from steps one and two, understanding ‘real life’ challenges for patients, health care professionals and health systems, was considered essential to inform an implementable intervention. We therefore conducted exploratory qualitative work with both health care professionals and patients with topic guides that addressed three issues:

i) Difficulties in living with COPD
ii) Opinions on a preliminary outline intervention (developed in step two)
iii) Critical elements for successful implementation

We identified respiratory health care professionals (rHCPs) who had an interest in delivery of psychological interventions to patients with COPD, through social media and professional networks, and invited them to participate in either an individual interview (face to face or telephone) or a focus group, dependent on participant preference.

We arranged two focus groups for patients and carers. One included COPD patients or carers who had previously experienced CBT (including participants from a specific CBT trial [39]; and one with patients and carers attending a Breathe Easy group (a UK support group run by the British Lung Foundation (BLF)) who may, or may not, have experienced CBT. Our PPI expert supported us in approaching and designing these focus groups and interpretation of the results.

Informed consent to participate and audio-record data was obtained for all participants. Recordings were then transcribed. As the purpose of this qualitative work was to inform intervention development, we conducted a rapid thematic analysis using a framework approach [40], with the key aim of identifying guiding principles that should be included in the intervention to increase acceptability and ease implementation into routine service if shown to be effective.

### Step four—Detailed design of intervention materials and mode of delivery

KHM and LS reviewed all feedback on the outline intervention and issues around delivery from step three and the guiding principles for the intervention and its implementation (see supplementary material 2). These were used to develop more detailed content of the intervention. In addition, at this stage, we were guided by recommendations for enhancing fidelity in behavioural interventions [41].

A reflective intervention development log was kept to ensure the process, considerations and decisions taken were recorded and transparent (see supplementary material 2).

### Step five—Whole intervention pre-pilot testing

Given the complexity and multi-level action of the TANDEM intervention, it was felt that even after individual materials had been developed and refined the intervention needed to be delivered as a whole, to understand how elements ‘hung’ together. This was seen as an explicit part of the design phase of the intervention, and an important step prior to testing any research elements (such as randomisation, or outcome data collection) that was scheduled for a pilot trial [42]. We identified two key questions which needed to be answered before formal piloting:

i) Do participants who receive the intervention when delivered by someone already skilled in CBT find it acceptable (i.e. is the potential TANDEM patient facing intervention appropriate and acceptable) and receive the intervention as intended (delivered with fidelity)?
ii) Do participants who receive the intervention when delivered by a novice in a cognitive behavioural approach (CBA) but trained as part of the TANDEM programme, find the intervention acceptable and receive it as intended (i.e. is the proposed TANDEM facilitator training sufficient for delivery of the TANDEM patient intervention and consequently implementable)?

A purposive sample of three rHCPs were recruited and trained to deliver the TANDEM intervention; these included one rHCP trained to CBT diploma level, one trained to basic CBT level (i.e. following a 3-day training external to TANDEM) and one rHCP who had not previously received any CBT training. Once trained in the TANDEM intervention, these individuals were referred to as ‘TANDEM facilitators’.

As part of the facilitator-facing intervention, training for the TANDEM facilitators was conducted over 3 days, the first two concurrent and the third approximately 6 weeks later to enable practice of skills. To reflect the group nature of future training, clinical members of the research team also joined the three days training as participant observers. Training was delivered by a CBT qualified consultant respiratory nurse (KHM), a health psychologist (LS) and a consultant clinical psychologist (SSW). At the end of each training day, all participants were requested to provide verbal feedback on the content and process of training.

Patients with moderate to severe COPD were eligible for participation in the whole intervention pre-pilot phase. Patients received the full 6–8 TANDEM sessions and were invited to interview post-intervention. With participant consent, all intervention sessions were audio-recorded. Following delivery of the patient intervention, all three of the rHCP TANDEM facilitators were invited to interview. Semi-structured interviews were conducted and covered issues such as acceptability and benefit of training, any omissions or improvements, feasibility of delivering the intervention to patients and usefulness of intervention materials.

## Results

### Step one—Building an expert team

Dr. Karen Heslop Marshall, a clinical respiratory academic who had experience of delivering CBT in COPD, agreed to be part of the expert team, as did Professor Sally Singh, a pulmonary rehabilitation expert. Education for Health, a health education charity, was approached and agreed as did psychologists Dr. Liz Steed and Dr. Sarah Saqi-Waseem. Chris Warburton was an expert patient who joined the team and offered PPI consultation throughout. Methodologists included Moira Kelly and Ratna Sohanpal, who both had experience of working in the field of COPD.

This expert team proposed combining CBA and a supportive self-management intervention with exercise, provided by explicitly linking in to routine PR, in order to build upon an evidence-based service already embedded within the NHS. The team proposed building on two pre-developed interventions The Lung Manual [43] and the SPACE Manual [44] including use of their materials, e.g. SPACE materials as well as the full range of information leaflets for people with COPD and their carers from the British Lung Foundation to meet educational and self-management needs. This meant materials which had already had extensive PPI input were available. We also drew on CORE competencies for delivering CBT [45].

### Step two—Use of theory to develop a logic model and preliminary outline of the intervention

Both The Lung Manual and the SPACE manual are evidence-based and draw on theory. The Lung Manual applies Beck’s theory of CBT [46] for managing anxiety and breathlessness in COPD whilst the SPACE manual applies a self-management approach based on Bandura’s social learning/cognitive theory [47, 48]. These theories were therefore considered important underpinnings for TANDEM. LS suggested that Leventhal’s Self-Regulation theory [49] may also be relevant to self-managing COPD. This was supported by the literature and therefore incorporated in the programme theory for patient-facing aspects of the intervention.

Consultation with Education for Health suggested that the pedagogical theory that would be most relevant for training facilitators was the VARK (Visual, Auditory, Read, Kinesthetic (i.e. experience or practice, simulated or real)) model of learning [50]. This has been used frequently in training interventions and ensures that the training suits individuals with different learning styles and we adopted it in the current study.

For intervention development, the person-based approach [51] with its focus on using qualitative work to inform guiding principles was considered particularly relevant and therefore guided the intervention development process.

**Fig. 2:**
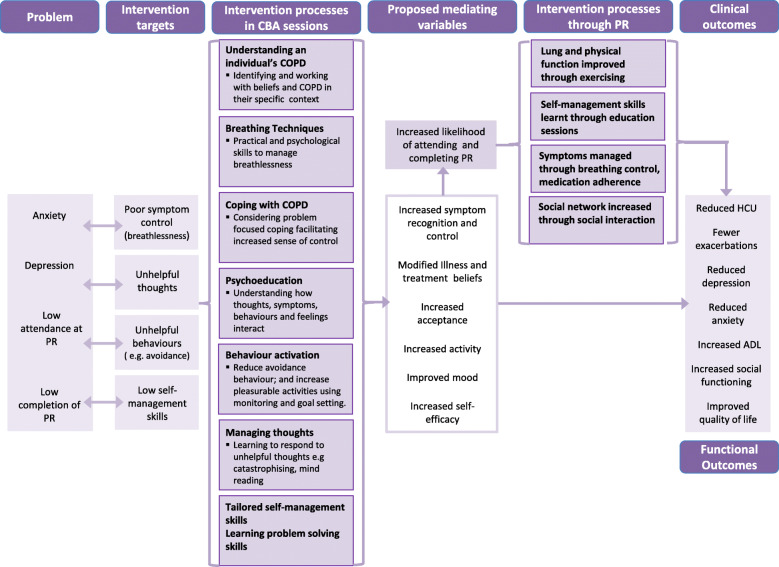
Logic model for TANDEM Intervention

Figure 2 shows the logic model for TANDEM. The basic premise is that how an individual thinks about their COPD (cognitions—including illness and treatment beliefs) influences how they behave (including self-management actions taken) and how they feel (both physical symptoms and emotions). These factors have interactional effects such that depression and/or anxiety can be both reduced or exacerbated depending on the individual’s cognitions and behaviours. Consequently, by targeting change at a cognitive, behavioural or symptom level, this will influence emotional outcomes. This improvement in emotional outcomes and self-management outcomes is then hypothesised to make attendance at pulmonary rehabilitation more likely, which itself is known to have positive outcomes including on both physical and psychological outcomes [30, 31].

Based on the logic model and content from the Lung and SPACE manuals, a preliminary outline intervention for discussion in step three was developed (see supplementary file one). This included information on COPD including illness and treatment beliefs and skills such as breathing techniques; cognitive behavioural techniques such as monitoring, diaries and distraction; and basic self-management techniques such as goal setting and problem solving; delivery options were open as were the best ways to train facilitators and who those facilitators should be and other factors which would support implementation.

### Step three—Qualitative findings, themes and development of guiding principles

One focus group comprising six rHCPs (one respiratory consultant, one occupational therapist, three physiotherapists and one exercise practitioner) and seven individual interviews (four psychologists, two physiotherapists and one general practitioner) were conducted. All participants had experience in working with patients with COPD, either in the community or secondary care. Roles varied, including some with management responsibilities who were able to discuss implementation.

One focus group was held with patients of whom four had COPD, two had other respiratory conditions and two were carers. Very tight timelines precluded formal analysis of transcripts from the patient focus group data, so data were limited to quotes selected from the audio recording. Major local governance delays prevented conduct of the second focus group with patients and their carers who had experience of CBT in time to inform intervention development.

Both patients and professionals presented an overall positive attitude to the idea of the TANDEM intervention:

And I do think in the long run something like this could be more cost effective and stuff, things like that … I think it would be really useful (rHCP FG002 Occupational therapist).

Themes were developed which related to (i) life with COPD, (ii) intervention considerations and (iii) issues for implementation.

#### Life with COPD

All participants, including patients and rHCPs, recognised depression and anxiety as common in COPD although patients did not always use this terminology. Other issues such as frustration and embarrassment, along with role adjustment and loss, including of social contacts, were common and seen as contributors to mood problems.

‘The approach is good … because of all the other things happening in people’s lives which can stop them attending PR and make them anxious and depressed.’ (focus group patient).

#### Intervention considerations

rHCPs emphasised the challenge of breathlessness to patients with COPD and suggested that discussion and teaching of breathing control early in the intervention would be a helpful way to raise issues around mood as well as providing practical help which may increase engagement. There was also recognition that this group may be quite socially isolated and health literacy may vary so the intervention must be accessible to all.

#### Implementation considerations

The majority of issues for implementation related to the workforce and who would realistically be able to deliver a CBA service. Both rHCPs and psychologists recommended rHCPs to be best placed, however all recognised that there would need to be some selection process and supervision. One clinician was concerned about the cost of the intervention, although others considered that in the long run CBA could be cost-effective.

These themes were subsequently interpreted to provide guiding principles as recommended by the patient-based approach to intervention development [51]. Table 1 shows these principles and example data extracts.

**Table 1:** Guiding principles for TANDEM

	Illustrating quotes
**Intervention guiding principles**	
Depression and anxiety are key topics but could be introduced via breathlessness	*I think they most often talk about symptoms like breathlessness, rather than saying that they are anxious or depressed. (HCP006 Physio)* *Terminology is important such as ‘dealing with’ ‘living with’ (patient)*
The Intervention should be tailored/flexible to individuals	*It’s just that patients are all different, and therefore present very differently and the intervention has to be tailored individually to what they are presenting with. (HCP003, psychologist)*
Sessions could be offered at home, or clinic but there may be limitations to the latter, accessibility is key	*So I think having the capacity to start off at home is certainly a good idea. I think just something about accessible locations*. *(HCP002, Psychologist)*
Clear expectations and boundaries should be set at the start of the intervention	*So there needs to be quite clear boundaries about what the intervention offers and does not offer.(HCP006, physio)*
**Implementation guiding principles**	
Delivery by respiratory professionals rather than psychologists is preferable	*It feels important that other members of the healthcare team are being trained up in these approaches. That can only be a good thing*… *(HCP003,psychologist)*
Some selection and training of facilitators will be needed	*A lot of people would be attracted to this, but it’s not for everyone to deliver.(HCP005, physio)* *What training would this nurse have? (Patient)*
Supervision of facilitators delivering the intervention is essential and should be ongoing	*I think that’s important.[supervision] (HCP005, physiotherapist)*
The intervention must be deliverable and supported by management	There’s no point evaluating it if it’s not something that’s going to be deliverable. *(HCP FG001 Doctor)*
The intervention should be able to account for patient breaks due to illness	*...that’s important, and you have to acknowledge if they are not feeling well, we have to assess it and make sure that they get the right treatment.* *(HCP005, Physiotherapist)* *Timing is important’(patient)*

### Step four: Detailed design of intervention materials and mode of delivery

Having agreed the guiding principles for the intervention the expert team met to discuss the detailed design of the TANDEM intervention. It was understood that the intervention would be working at two levels: (i) patient-facing (i.e. CBA delivery) and (ii) facilitator-facing (i.e. training programme).

For the patient-facing CBA a range of core patient self-completion materials were designed that could be provided as part of the intervention. These covered the topics of ‘Controlling your breathing’, ‘Mood and COPD’, ‘Anxiety and COPD’, ‘Depression and COPD’, ‘Problem Solving’ and ‘Saving Energy’. These were developed for TANDEM but where possible drew on, or used, the SPACE manual [45] handouts and were of similar format to published CBT leaflets accessible on the internet [52] as these have been developed with extensive PPI. The aim of these leaflets was as homework (called ‘home practice’ in the TANDEM intervention based on PPI advice that connotations of school may be off putting for people who had poor experiences of school), which is a central part of CBT and to reinforce knowledge that had been covered in the one-to-one sessions. The approach is also in line with Integrated Access to Psychological Therapies (IAPT) services low-intensity provision [53]. LS developed all materials with iterative refinement from the expert team including PPI. At the start of session one, each patient was given a TANDEM folder, in which they could store handouts relevant to them so that individuals had a tailored version of TANDEM materials whilst maintaining consistency in the content provided. In total, 6–8 face to face sessions were designed, covering nine topics, with core content and additional modules tailored to individual problems and complexity. Table 2 provides an overview of TANDEM topics.

**Table 2:** Summary of TANDEM intervention (patient facing) content

	*Topics covered*	*Content*
*Session 1*	Introduction, setting expectations **Topic 1**—What is COPD? **Topic 2**—Taking control of COPD **Topic 3**—The patient experience of breathlessness	Eliciting the patients understanding of COPD, identifying and working with illness and treatment beliefs and acceptance. Teaching basic breathing control.
*Session 2*	Feedback from home practice **Topic 4**—Introducing mood and COPD	Conducting a formulation and presentation of a cognitive behavioural approach
*Sessions 3–7*	Feedback from home practice **Topic 5**—Managing anxiety and COPD **Topic 6**—Managing depression and COPD **Topic 7**—Applying the CBA to other problems (optional)	Up to four sessions to conduct cognitive behavioural work on anxiety and/or depression dependent on individual need. One further session available to discuss other problems if needed.
*Sessions 5–7*	Feedback from home practice **Topic 8**—Living with COPD day to day	Self-management approaches to COPD. Learning to problem solve and set goals.
*Sessions 6–8*	Feedback from home practice **Topic 9**—Preparing for pulmonary rehabilitation	Expectations of PR, addressing worries and concerns

One topic (dealing with ‘other problems’) was specifically added as a strategy for keeping the focus of initial sessions on COPD whilst having space later to address issues the person may have outside COPD, for example debt, substance abuse etc. This topic looked at how the CBA, learnt in the context of COPD, could be generalised to different problems, with sign-posting to additional sources of help. At the final session, discussion was around pulmonary rehabilitation. If there was to be a delay in individuals commencing PR, then the facilitator arranged up to nine weekly telephone calls.

The 3-day facilitator training was provided with a supporting manual which covered the skills needed to deliver TANDEM.

Table 3 shows the content of the training programme. There was a high level of practical and experiential learning in the group and supportive links within the group were encouraged. Throughout delivery of the CBA intervention, facilitators received regular telephone supervision, one-to-one with senior cognitive behavioural therapists at approximately fortnightly intervals. This on-going supervision was considered to be an integral part of the intervention.

**Table 3:** Overview of TANDEM facilitator training

Day 1	Day 2	Day 3
• Introductions • **TANDEM overview** • The patients experience of COPD (group exercise) • What are depression and anxiety? (group exercise) • Depression and anxiety in COPD • Introduction to CBA (group exercise) • Core therapeutic skills (**video demonstration**) • Making an assessment—recognising thoughts, feelings, behaviours, symptoms (practical) • Sharing ideas with patients (practical) • Feedback on worries and concerns after day one	• CBA techniques (practical) ◦ Psychoeducation ◦ Breathing control ◦ Distraction ◦ Monitoring ◦ Problem Solving ◦ Goal setting ◦ Graded practice/simple behavioural experiments ◦ Challenging thoughts **(video demonstration)** • **Toolbox for anxiety** • **Toolbox for depression** • Preparation for case studies	• Case study feedback • **Individual practice with actor (videoed)** • Delivering TANDEM session by session including ◦ Changing behaviour ◦ Preparing for PR (using a photobook) • **Importance of supervision** • Risk assessment • Research requirements **Provision of crib cards**

Bold typeface represents additions to the training after conducting the real world pre-pilot

### Phase five—Whole intervention pre-pilot study

All three TANDEM facilitators completed the three training days and two went on to deliver the intervention to three patients (one delivered it to two patients and one to a single patient). The third facilitator (a respiratory practice nurse without prior CBT training) did not manage to see any patients due to unanticipated research governance delays and a consequent change in work commitments.

#### TANDEM facilitators

After the initial training session, facilitators suggested some changes (see supplementary file two), specifically an overview of the intervention at the beginning of the training in order to orientate individuals. All the facilitators felt that the role-play activity with a simulated patient (actor), which was conducted as part of the original group training on day 1, was too threatening and at too early a stage of skill development. Instead, they requested more demonstrations and more practice in developing a formulation.

Interviews at the end of delivering TANDEM to patients revealed that both facilitators felt the intervention had been well received by participants and feasible to implement, although one had to deliver it over a longer period than scheduled due to patient illness.

‘Yeah, I mean the two patients who I had were very, very enthusiastic about all elements of the intervention. (PPHCP01)’

Generally, the facilitators appeared able to follow the manual and found it a helpful guide, but there was questioning of whether someone without previous CBA training would be able to manage:

‘I mean section nine, it’s got identifying maintenance factors, and it talks about safety behaviours, avoidance and escape, catastrophic interpretation, scanning or hypervigilance, self-fulfilling prophecies, fear of fear, reductions, affectionism, short term rewards. If you’re trying to talk to a patient and remember what it says in the manual you might get yourself a little bit flustered.’ (PPHCP02)

One facilitator recommended presenting basic intervention techniques as a toolbox and also the provision of a crib sheet for easy prompting within sessions.

‘I feel that people who come away from the training need to have something like a virtual toolbox of techniques that they can refer to … they expected quite a lot of you … I made myself a crib sheet type of thing’ (PPHCP01)

One element that was not adhered to as planned was supervision with a senior psychologist, as the facilitators relied on supervision by an experienced member of their team who was already known to them and who was also part of the study team (KHM). However, both facilitators reported this supervision was useful.

#### Patient perspective

Patients who had received the TANDEM intervention reported it to be acceptable and beneficial, observing that the facilitators had very good interpersonal skills. There were no substantive suggestions for improvement.

‘And then [facilitator] and I just seemed to get on very well, he's a likeable chap, very laid back. And so it went from there. And then we started doing the things that you asked in TANDEM. Planning … They're just small things, but marvelous’ (PPP01, male participant)

The findings, such as patients reporting activities like planning (see quote above), and facilitators commenting on applicability of acceptance exercises suggested that the facilitators delivered the intervention with fidelity; however, this needs to be explored in more detail through use of for example audio-recordings of sessions. This is planned for the process evaluation in the pilot and main trial (Kelly M, SL, Sohanpal R, Pinnock H, Taylor S. The TANDEM trial: protocol for the process evaluation of a randomised trial of a complex intervention for anxiety or depression in people living with chronic obstructive pulmonary disease. Under review).

### Refinements to TANDEM after the pre-pilot

Changes and additions were primarily made to the TANDEM facilitator training, as there were few recommendations for changes to patient materials. These are shown in Table 3 with additions highlighted in bold. All suggestions were followed: e.g. providing a greater overview of TANDEM at the beginning of session one, keeping to a core set of CBA techniques and outlining a ‘toolbox’ of techniques which could be used (literally presented like a tool box in the revised manual). We made video recordings to demonstrate therapeutic skills and CBA techniques. These were made available online, with a facilitator chat facility for ongoing support.

The use of a simulated patient was omitted from the first 2 days of training and replaced by partnered role-play. The simulated patient role-play was, however, added to the end of day 3. Each TANDEM facilitator was individually video-recorded conducting a cognitive behavioural assessment and feedback with the actor. Each video was subsequently assessed by LS and at least one independent assessor with a psychology background to ensure that a minimum standard of competency (see study protocol [37]) had been acquired. This also enhanced fidelity of delivery. To enhance learning, and boost confidence, facilitators received one-to-one feedback on their video. A training session on the importance of supervision was added with reflection that supervision is a standard part of psychological training and practice (in contrast to more managerial supervision with which HCPs may be more familiar).

#### Refinements to improve implementation

To improve delivery of the intervention within the trial and future implementation within routine healthcare contexts, five features were added:

i) Facilitators were provided with crib cards for use as prompts within sessions.
ii) An optional session was added for use when a break in sessions had become necessary (e.g. due to patient illness) in order to refresh topics that had been covered before the break and re-establish current priorities.
iii) Some flexibility in the order of delivery of sessions was allowed reflecting the reality that some patients commenced PR before the end of the TANDEM sessions. It was stipulated that Topics 1–5 (or 6) must have been conducted but that if necessary the final topic on expectations of PR could be brought forward as there was no sense in delivering this once PR had started.
iv) A structure for screening potential facilitators including a formal application with a curriculum vitae and telephone interview with one of the principal investigators was developed. The aim was to ensure only fully committed individuals who were interested in the psychological aspects of the experience of living with COPD and who could meet the study-specific requirements (e.g. flexibility to travel, willingness to complete research modules and good clinical practice training) received training. Also facilitators needed to be made aware that training involved some role play and that all intervention sessions would be recorded for fidelity assessments.
v) A booster training session was designed to be delivered to facilitators if there were delays of 3 months or more between initial training and delivery of TANDEM.

For a description of the intervention following TIDieR guidance, please see Additional file 1.

## Discussion

This paper describes the development of the TANDEM intervention for COPD that considers both intervention and implementation strategies from the outset, with the aim of reducing the time period for translation of the intervention (if successful) into practice. In particular, consideration was given to the workforce that would deliver the intervention and their training and support needs, as well as designing an intervention that could fit structurally into routine clinical care. This would also make it more likely the intervention could be delivered with fidelity.

An important element of development was the whole intervention pre-pilot testing. This is particularly applicable for complex multi-level or group interventions where the recommended ‘think aloud’ phase [51] of development may be impractical. The benefits of a pre-pilot phase are to test not only individual elements of an intervention but also how multiple elements of an intervention ‘hang together’. Similar recommendations have been made by others such as in the ORBIT Model [54]. Thorough testing in the development phase is important and should come before a pilot trial which can then focus more on testing elements of the research process [42]. Further testing of acceptability can still be done when piloting the intervention, as is planned for the TANDEM intervention (Kelly M, SL, Sohanpal R, Pinnock H, Taylor S. The TANDEM trial: protocol for the process evaluation of a randomised trial of a complex intervention for anxiety or depression in people living with chronic obstructive pulmonary disease. Under review); however, the fewer changes that need to be made further along the evaluative trajectory the more efficient evaluation of the intervention is likely to be.

Whilst we present a stepped process in reality, there was flow between different steps as intervention development is commonly iterative [2]. For example, a preliminary outline of the intervention had been developed in step two before the guiding principles were articulated in step three. This was inevitable as our initial design was reactive to a funding call (with the additional benefit of enabling peer review comment on our ideas). Importantly, however, at step three, we were open to any of our original ideas being challenged and the intervention changed. The expert study team comprised individuals with diverse backgrounds and were encouraged to reflect and discuss critically.

A challenge we recognised from the outset was that for interventions to be implemented within routine health care services they must be as cost effective as possible. One implication of this is that each patient should receive the optimal intervention for them; one size fits all interventions are likely to be cost inefficient either by under or over treating some individuals. Tailoring has been recommended as a way to address this [7], though it is a significant challenge to design an intervention that is both responsive to each individual’s needs yet standardised enough to allow for robust evaluation and assessment of fidelity when implemented. TANDEM attempted to balance standardisation with flexibility by using a modular/topic-based approach where topics are standardised, but which topics are addressed and in what order is dependent on the individual or the circumstances (such as timing of the PR course). The success of tailoring in this way is as yet unknown but will be examined as part of the process evaluation of the trial as will fidelity.

In order to maximise fidelity, we required an interview, completion of the full training programme and demonstration of post-training skills before approval as a TANDEM facilitator. It will be of interest whether this vigorous approach has an impact on the uptake of TANDEM within routine care, for example whether members of the pulmonary rehabilitation team will be prepared and able to undergo this level of training, although this was recommended by health care professionals during initial qualitative work.

A challenge for the current study was that some aspects of implementation were more difficult because of delivery within the context of a research trial. In our pre-pilot, we experienced two illustrations of this. The first was that obtaining local research governance approval caused a delay of 6 months between training TANDEM facilitators and being allowed to deliver the intervention (arising when a local research governance office unexpectedly changed its classification system). This was detrimental as it risked loss of confidence among the facilitators, skill drift and knowledge decrease during the gap between training and implementation. We would urge research governance frameworks to consider the impact of their processes in the context of intervention development and implementation research. Within a clinical service, this might be less likely to occur, but clinical practice has its own challenges such as staff turnover, funding pressures and changes in management. The implementation strategies we provided, such as booster training and online support resources to overcome study specific issues, could equally apply in the clinical setting and will be explored within our trial process evaluation.

The second was that our TANDEM facilitators could not be embedded within the participants’ clinical team (to prevent ‘contamination’ of controls [37]) with the results that they would not have support, both practical and emotional, from clinical colleagues. The research team attempted to overcome this by providing facilitators with a clinical support network with the chief investigators ST, HP (both general practitioners) and the consultant respiratory nurse (KHM) available to answer any concerns. This was in addition to the clinical supervision provided for ensuring therapeutic competence and access to a chat facility with other facilitators on the skills website.

### Strengths and limitations

A strength of TANDEM is in its multidisciplinary expert team approach to intervention development with PPI representation within that team so embedded throughout all our work. We specifically built upon previous work by collaborating with national experts enhancing the expertise available to TANDEM and reducing potential duplication. Surprisingly, this has not been explicitly recommended in recent guidance [11] but in our view is an important strategy for ensuring the most efficient use of limited resources.

A further strength is the systematic and transparent approach to intervention development that we have outlined.

In step three where we conducted qualitative work to highlight important issues for the intervention and its implementation, only a limited number of health care professionals and patients could participate. It may be that important voices such as that of practice nurses, health service managers or commissioners, who could have provided different perspectives for implementation, were not heard. This step, however, was scheduled within a relatively rapid time-frame to be useful to inform intervention development, and the themes will be further explored in the pilot/trial process evaluation.

In the pre-pilot phase, only three facilitators and three participants experienced the intervention. Greater numbers would have provided greater feedback; however, again, this was not possible due to resource limitations and logistical requirements of the time-scale required for intervention development. Similarly, due to time constraints, we were also not able to fully evaluate fidelity; however, this is planned for the pilot and main trial evaluations (Kelly M, SL, Sohanpal R, Pinnock H, Taylor S. The TANDEM trial: protocol for the process evaluation of a randomised trial of a complex intervention for anxiety or depression in people living with chronicobstructive pulmonary disease. Under review) and is an important part of intervention development.

Although we aimed to address integration of implementation issues throughout our intervention development process, we did not explicitly do this in our research design and hence have not presented a logic model of implementation which will be presented following process evaluation. Methodological advances in implementation research have made recommendations for trial designs known as hybrid designs whereby effectiveness and implementation potential are investigated concurrently [38]. Recently, there have also been calls for this within behavioural science [55]. Three levels of hybrid design are proposed which vary in the relative balance between the focus on effectiveness versus implementation [40]. These designs require a set of assumptions to be met around features of the intervention, such as the level of face validity, strength of the existing evidence base, risks associated with the intervention and implementation momentum. TANDEM meets these assumptions and hence can be considered as similar to a type one hybrid design where the primary focus is on effectiveness but issues of implementation are explored for example:

i) It has high face validity given CBT and pulmonary rehabilitation are well recognised and guideline-recommended interventions.
ii) PR has an established evidence base for COPD [29–31] and CBT is showing promise for COPD patients [35, 42].
iii) Few risks have been described in evaluations of CBT or PR and there is little reason to expect increased risk through the integration of these approaches in TANDEM;
iv) Importantly, there is currently momentum within the clinical system towards both implementation of PR [56], and treatment of psychological issues within chronic illness, in the UK [57]. Furthermore, recent policy has advocated the expansion of current primary care mental health services to work with patients with COPD (IAPT services) [53].

Future studies developing interventions for implementation may wish explicitly to consider a greater diversity of research design, including hybrid designs to facilitate implementation.

## Conclusions

We recommend that intervention developers play greater consideration to implementation issues both at the early stages and throughout the intervention development phase. We describe five steps including building an expert team and building on previous innovations, using theory, exploring the needs of the target groups, developing prototypes and testing the whole intervention in a pre-pilot. By conducting this work within a framework of critical reflectivity, we aimed to maximise efficiency of intervention design and minimise the trajectory from intervention development to implementation if shown to be effective.

## Supplementary Information

**Additional file 1.** TIDieR Framework.

**Additional file 2.** GUIDED Checklist.

**Additional file 3: Supplementary File 1.** Outline of the intervention developed in step two and presented at step three.

**Additional file 4: Supplementary File 2.** Feedback and amendments made throughout the intervention development process (step 4 & 5).

## Data Availability

Data generated and analysed during this study is included in this published article. Study manuals and training materials are copyrighted but will be available upon reasonable request to the authors.

## Abbreviations

BLF: British Lung Foundation
CBA: Cognitive behavioural approach
CBT: Cognitive behaviour therapy
COPD: Chronic obstructive pulmonary disease
GUIDED: GUIDance for the rEporting of intervention Development
IAPT: Integrated Access to Psychological Therapies
MRC: Medical Research Council
PPI: Patient and public involvement
PR: Pulmonary rehabilitation
rHCP: Respiratory health care professional
TANDEM: Tailored intervention for ANxiety and DEpression Management
TIDieR: Template for Intervention Description and Replication
VARK: Visual Auditory Reading Kinesthetic
WHO: World Health Organization
WIDER: Workgroup for Intervention Development and Evaluation Research
