# Supplementary material for: Developing a complex intervention whilst considering implementation: the TANDEM (Tailored intervention for ANxiety and DEpression Management) intervention for patients with chronic obstructive pulmonary disease (COPD)
Source: Trials. 2021 Apr 6;22:252. doi: 10.1186/s13063-021-05203-x (PMC8025339; doi:10.1186/s13063-021-05203-x)
Supplement: Supplementary file 1 — Additional file 1. TIDieR Framework. [file 13063_2021_5203_MOESM1_ESM.docx]

Supplementary File 1: TIDieR Description of TANDEM

| 1. Brief name | TANDEM |
| --- | --- |
| 2. Why | Uptake and retention to pulmonary rehabilitation in COPD is sub-optimal. One reason for this may be the co-occurrence of anxiety and or depression in addition to their physical illness. By targeting an intervention at anxiety and depression pre-attendance at PR it was hoped that there would be increased psychological well-being and increased uptake and retention in PR services as compared to a usual treatment control.  The current cognitive behavioural approach (CBA) intervention draws on the principles of CBT (44) as well as practical self-management skills drawn from the SPACE COPD manual (43) and Leventhal’s common sense model of illness regulation (47). Health care professional training was provided to enable delivery of the patient intervention and this additionally drew on pedagogical principles (VARK) (48). |
| 3. What | Materials:  Patient facing intervention: As part of the study participants were provided with the British Lung Foundation (BLF) DVD and Booklets <http://shop.blf.org.uk/collections/copd>  - Living well with COPD  - First steps to living with COPD booklet (code BK31)  - Pulmonary rehabilitation and exercise booklet (code BK27)  (N.B this was also provided to the control group).  In addition a range of 11 self-completion leaflets designed specifically for this trial were available. Seven of this were core leaflets which all participants received and a further four leaflets were provided as required. In addition all handouts from the SPACE manual were available for facilitators to hand out as required and BLF leaflets  - Sex and breathlessness (code FL22)  - Going on Holiday with a lung condition (code BK6)  - Get self-help leaflets for psychosocial difficulties  Facilitators also had access to a range of resources for use in sessions such as formulation sheets, activity and thought diaries.  Carers were given - The BLF leaflet: Looking after someone with a lung condition booklet (code BK21) if appropriate.  Facilitator training: A range of materials were used during training including powerpoint slides, individual and group exercises, case studies, video demonstrations, participant presentations, *video-feedback.* Training was supported by a TANDEM manual covering the topics addressed during training, *crib cards and* *access to a website with uploaded skills videos and a chat function with other facilitators.* The training providers followed a training guidance document and used standardized powerpoint slides and videos to demonstrate skills.  *Supervision training: Supervisors were provided with a half day training supported by powerpoint presentation and group discussion and provided with copies of the TANDEM manual.* |
|  | Procedure  Patient facing intervention: Following recruitment and completion of baseline assessments participants in the intervention group were contacted by their allocated facilitator and an appointment made for the initial session. Subsequent sessions were arranged at the end of each session or through telephone contact. The aim was for sessions to be weekly, however flexibility was allowed where required, particularly for health reasons  *Facilitator training: Facilitators were recruited through advertisements for the position. Interested individuals were interviewed over the telephone by PIs to ensure appropriate qualifications and fit with the role.*  *Assessment of CBT skills was conducted on day three of the training by video with a professional actor. Only individuals acquiring >27 on the CFARS scale (37) were invited to be facilitators*. Trainees who did not meet the required standard were offered additional training at their own cost if desired.  Supervision: Prior to commencing supervision supervisors were invited to attend the TANDEM facilitator training and then received an additional half day training outlining expectations and skills needed for the role. |
| 5. Who Provided | Patient Facing: Respiratory professionals (including nurses, physiotherapists, occupational therapists, and health psychologists) who had attended the TANDEM training and acquired at least the minimum level of proficiency (CFARS score 27) delivered the one to one in person sessions with patients. These facilitators also delivered telephone sessions before and during PR to ensure a seamless intervention.  Facilitator Training: A Health Psychologist and a Consultant Respiratory nurse trained in CBT delivered day one and two of the training. A Clinical Psychologist and the Health Psychologist delivered day three of the training. The PI conducted the introduction to the training programme. The health psychologist delivered face to face feedback on the video role play assessment. A clinical psychologist delivered supervision to facilitators throughout intervention delivery.  Supervisor Training:- Training was delivered by the PI and clinical psychologist. |
| 6. How | Patient Facing Intervention: The CBT sessions were primarily delivered face to face, and individually (or with partner/carer present if desired). PR support sessions were delivered over the telephone. N.B As a consequence of the CoVID outbreak 2020 some of the final intervention sessions had to be delivered by telephone.  Facilitator training: Training days 1-3 were delivered face to face as a group. Feedback on the video assessment was delivered individually face to face.  Supervisor training: Face to face in a group  Supervision: Mode of delivery was dependent on facilitator choice but in the majority of cases was by telephone. Group peer supervision was offered online with moderator input. |
| 7. Where | Patient Facing Intervention: Delivered in the patients preferred location including home, GP surgery, hospital clinic.  Facilitator Training: Training was generally delivered at Queen Mary University of London. Feedback to individual facilitators on video role-play was conducted at the facilitators’ place of work or location of choice.  Supervision: Delivered by telephone or remote video call |
| 8. When and How much | Patient Facing Intervention: Following confirmation of eligibility the first TANDEM session was arranged. Sessions ideally took place weekly for approximately 40 minutes. Depending on the individuals need they received between 6-9 sessions. Following this, participants are given a date to commence routine pulmonary rehabilitation (PR) at their local service. In the gap between the intervention being finished and pulmonary rehabilitation commencing participants receive one to one phone calls by the TANDEM trained health care professional on a weekly basis (duration of each call 10-15 minutes). Weekly phone calls continue whilst the participant is attending PR and for 2 weeks after the completion of PR.  Facilitator Training: Facilitator training was planned to take place as close as possible to intervention delivery, however in many instances there was some delay. The training was delivered over 3 whole days. Days 1 and 2 were offered concurrently with day 3 approximately 4-6 weeks later. The aim was for day 3 training to be offered as close to intervention delivery as possible.  Supervisor Training: One half day training was delivered.  Supervision: Thirty minute supervision sessions were organized approximately fortnightly although supervisors were available for urgent queries outside of these meetings if necessary. |
| 9. Tailoring | The patient intervention was tailored for individual by determining primary presenting problem (anxiety or depression) and addressing this first. Number of sessions of CBT was also tailored to individual depending on complexity of issues and individual progress.  Facilitator training was not specifically tailored, although all training was responsive to the needs of individual group members and supervision was tailored to the individual facilitators needs.  Supervisor training was not specifically tailored to supervisor, however it was responsive to participants queries and needs. |
| 10. Modifications | Modifications made post-pre pilot are highlighted in italics. Due to the Covid outbreak in March 2020 any remaining TANDEM face to face patient sessions had to be delivered by remote means including telephone or videoconferencing. |
| 11. How well | We aimed to increase intervention fidelity by selection of facilitators who demonstrated competence at the end of training, as well as by providing ongoing clinical supervision by a clinical psychologist. All TANDEM sessions are being audio-recorded and rated according to a coding system by an independent coder. |
| 12. | Fidelity assessment is ongoing and will be evaluated as part of the process evaluation. |
