# Supplementary material for: Developing a complex intervention whilst considering implementation: the TANDEM (Tailored intervention for ANxiety and DEpression Management) intervention for patients with chronic obstructive pulmonary disease (COPD)
Source: Trials. 2021 Apr 6;22:252. doi: 10.1186/s13063-021-05203-x (PMC8025339; doi:10.1186/s13063-021-05203-x)
Supplement: Supplementary file 3 — Additional file 3: Supplementary File 1. Outline of the intervention developed in step two and presented at step three. [file 13063_2021_5203_MOESM3_ESM.docx]

Supplementary Material 1 – Outline of the intervention developed in step two and presented at step three

The outline intervention was developed by LS and KMH by drawing on resources in the Lung Manual (44), the Space Manual (45) and CORE competencies for delivering CBT (46 . The intervention outline was presented at step three in a 15 minute presentation and addressed the following:-

| - Introduce depression and anxiety as prevalent in COPD - Introduce aim of TANDEM – i.e. to reduce anxiety and depression and increase attendance at Pulmonary Rehabilitation - Discuss number of sessions e.g. 6-8 sessions with a trained HCP - Discuss length of sessions ~ 40 minutes in patients’ home or clinic - Show common vicious cycle of anxiety and depression in COPD - Mention example cognitive behavioral exercises e.g. activity diaries, goal setting, thought challenging - Present 8 proposed topics in TANDEM   - T1 - What is COPD?   - T2 - Taking control of COPD   - T3 - The patient experience of breathlessness   - T4 – Introducing mood and COPD   - T5/T6 – Anxiety/Depression   - T7 - Living with COPD day to day   - T8 – Preparing for PR - Present HCP training   - 2 days initial training in CBA techniques   - 6-8 weeks practice with telephone supervision   - 1 day follow up with case presentation & how to deliver TANDEM   - Training manual with structure of sessions   - Weekly Supervision - Questions   - Do you think patients will find this acceptable?   - Are we missing key topics?   - What will be challenges?   - If you were delivering/receiving what would you want support with? |
| --- |
