# Supplementary material for: Developing a complex intervention whilst considering implementation: the TANDEM (Tailored intervention for ANxiety and DEpression Management) intervention for patients with chronic obstructive pulmonary disease (COPD)
Source: Trials. 2021 Apr 6;22:252. doi: 10.1186/s13063-021-05203-x (PMC8025339; doi:10.1186/s13063-021-05203-x)
Supplement: Supplementary file 4 — Additional file 4: Supplementary File 2. Feedback and amendments made throughout the intervention development process (step 4 & 5). [file 13063_2021_5203_MOESM4_ESM.docx]

Supplementary material 2: Feedback and amendments made throughout the intervention development process (step 4 & 5)

|  | Feedback | From  Whom | Impact on Intervention |
| --- | --- | --- | --- |
| **Feedback following focus groups and interviews (Step 3)** | | | |
| Patient Facing Content | Frustration and embarrassment are important issues | rHCP | Frustration and embarrassment added to HCP training as emotions to explore within depression and anxiety |
|  | All patient materials need to be COPD specific | PPI | TANDEM specific self-help leaflets developed for mood and COPD, Depression and COPD, Anxiety and COPD etc |
|  | Breathlessness is perhaps the most important topic for patients and introducing this early is a good approach. Also distinguishing between good and bad breathlessness is important. | rHCP & PPI | Breathlessness addressed within the first session and as a ‘way in’ to mood and COPD.  How to rate your breathlessness and what to aim for also added. |
|  | In general a good intervention which is likely to be acceptable to patients | rHCP & PPI | No change |
| Delivering the intervention | If patients have problems other than COPD it can make it difficult to stay on track | HCP | An additional topic ‘Applying CBT to other topics’ was added as a strategy to acknowledge patients’ agenda and help facilitators keep focused on COPD in the initial sessions of the intervention. Signposting where appropriate would then take place with provision of materials as appropriate (E.g. from [www.ntw.nhs.uk](http://www.ntw.nhs.uk) or [www.getselfhelp.co.uk](http://www.getselfhelp.co.uk)) |
|  | Patients have varying health literacy and this should be addressed | HCP & PPI | The topic of health literacy and how to tailor the patient intervention according to this was added to the HCP training  All patients to be provided with BLF DVD which provides clear explanations of COPD |
|  | Carers may wish to attend the sessions  Carers may make sessions more difficult | PPI  rHCP | To give patients the choice on whether carers should attend session.  Develop a carer specific handout including details of ways in which support can be more or less helpful to the person with COPD |
|  | Facilitators should be respiratory professionals but selection and supervision necessary | rHCP | A selection process for respiratory professionals developed. Supervision already planned |
| **Feedback on v1 of TANDEM HCP Manual and Patient Handouts – verbal/written feedback (Step 4)** | | | |
| Manual Content | Suicide Risk Assessment and Management Plan needs to be added to manual | Clin Psych | Risk assessment and plan developed incorporating 24 hr contact numbers of PI GPs |
|  | All psychology needs to be in lay terms and suitable for non psychologists | Resp Nurse | Manual reviewed and simplified for non psychologist facilitators |
|  | The cognitive behavioural model of ‘Antecedents, behavior, consequences’ ABC is too complicated for non psychology facilitators | Resp Nurse | ABC model removed and focus just on hot cross bun model and vicious cycles of anxiety/depression |
|  | Benefits of exercise should be emphasized throughout intervention and examples should be COPD specific | Resp Nurse | All examples reviewed to be COPD specific and exercise reinforced throughout manual |
|  | Coping and the difference between proactive and passive coping is important | Clin Psyc | Discussion around coping added to the topic ‘Living with COPD day to day’ |
|  | Session overviews need to be placed at the beginning of the manual, this can be followed by how to deliver techniques then any background theory | Team | Manual ordered as suggested |
|  | It is important that facilitators know they are not using CBT just skills based on this approach | Clin Psych | Agreement to use the term CBA (cognitive behavioural approach) for the skills that TANDEM is using and highlighted in both manual and training that |
|  | Consider using icons to make manual user friendly | GP PI | Icons used to indicate where a question is being asked or an exercise to take place. |
| Patient Handouts | Add a jargon buster for patients | PPI | List of all acronyms and terms added to inside cover of all patient folders |
|  | Use more diagrams to explain what COPD is | PPI | More diagrams added to leaflets |
|  | More lay language needed, abstract ideas too complex | PPI | Language simplified in line with PPI recommendations. An example ‘the bang in the night’ removed |
|  | Photos of how to do breathing exercises may be helpful | RS | All SPACE patient materials which include photographs and have undergone extensive PPI review made available to patients as required |
|  | Guilt can be a strong and common emotion | PPI | How to manage guide addressed in facilitators training and added to patient leaflet on mood and COPD |
| **Feedback following pre-pilot delivery of the intervention (step 5)** | | | |
| Facilitator training | The training should start with an overview of all sessions to provide greater context | PP-F | Training revised as suggested |
|  | Need to add detail about how to use GAD-7, PHQ-9 | PP-F | Information added on why GAD-7, PHQ-9 are being used and how to administer them (also added to manual) |
|  | Having an actor to practice role play in front of others on day two is not acceptable. | PP-F | Use of a simulated patient removed from first two days training, but added to day three. Each facilitator has an opportunity to role play with the simulated patient in private. This is video-recorded and then feedback provided. |
|  | More time should be spent on how to develop a formulation | PP-F | More practice case studies added to develop formulation skills |
|  | More demonstration of skills requested | PP-F | Videos of all skills recorded and made accessible to facilitators |
|  | Greater cross referencing between training slides and manual would be helpful | PP-F | Slides and manual more comprehensively cross referenced |
|  | It would be helpful to have one case study that runs throughout training | PP-F | A case study TOM added to run throughout first two days |
| Facilitator Manual/Materials | Case example for acceptance should be COPD focused | PP-F | Acceptance case scenario changed from party scenario to COPD specific |
|  | Add materials needed for each session and aim of session | PP-F | Each topic starts with resources needed and objectives of topic |
|  | It would be helpful to list key issues to cover in each topic | PP-F | Key issues for each topic added to cover sheet of each topic |
|  | A crib sheet for use in sessions would be helpful | PP-F | Prompt cards developed for use in sessions and provided to all facilitators |
|  | Details on safety behavior should include overuse of inhaler | PP-F | Overuse of inhaler as a safety behavior added |
|  | More detail needed on how to score GAD-7, PHQ-9 | PP-F | Within training section added to use of GAD-7 and PHQ-9 with details on both scoring and interpretation added |
|  | Materials needed to support transition to PR | Team | A photobook was developed to show what can be expected from PR in the local area. Includes details on how to access PR. Shown and discussed in final session |
| Patient Handouts | The term homework is off putting with reminders of school | PPP | The term homework replaced with home practice |
|  | It can be helpful to have reminders to complete home practice | PPP | Reminders allowed at facilitator discretion |
| General | Health problems can cause breaks in delivery of sessions | Team | An additional session may be arranged when there has been a break for health reasons, this can be used to review changes since last meeting and revise previous content |
|  | In some areas PR may start before final session of CBA | Team | Final topic can be discussed early if needed but not before delivery of topics 1-5/6 which are considered minimal core of the intervention |
| Supervision | Supervision not used well | Team | Training should be clearer on the requirement of supervision and how psychology supervision may differ from previous experiences of supervision |
|  | 15 minutes insufficient time to discuss formulation | Team | Additional supervision time added (15 mins per case) for new cases at end of formulation |

rHCP – Respiratory Health Care Professional; PPI – Patient and Public Involvement; Clin-Pysch – Clinical Psychologist; PPF – Pre-pilot facilitator; PPP – Pre-pilot patient
